# Survey of sex/gender diversity in the GEDA 2019/2020-EHIS study – objectives, procedure and experiences

**DOI:** 10.25646/9958

**Published:** 2022-06-29

**Authors:** Kathleen Pöge, Alexander Rommel, Anne Starker, Franziska Prütz, Katharina Tolksdorf, Ilter Öztürk, Sarah Strasser, Sabine Born, Anke-Christine Saß

**Affiliations:** 1 Robert Koch Institute, Berlin, Department of Infectious Disease Epidemiology; 2 Robert Koch Institute, Berlin, Department of Epidemiology and Health Monitoring

**Keywords:** SEX/GENDER DIVERSITY, GENDER IDENTITY, GEDA/EHIS, HEALTH MONITORING

## Abstract

Sex/gender diversity is increasingly recognised by society and should be taken into account more in population-representative studies, as they are important data sources for targeting health promotion, prevention and care. In 2019, the Robert Koch Institute started a population-representative health survey with the study Health in Germany Update (GEDA 2019/2020-EHIS) with a modified, two-stage measures of sex/gender. The survey covered sex registered at birth and gender identity with an open response option. This article describes the aims, the procedure and the experiences with the operationalisation of sex/gender and the results. Out of 23,001 respondents, 22,826 persons are classified as cisgender, 113 persons as transgender and 29 persons as gender-diverse. 33 respondents were counted as having missing values. A survey of interviewers showed that the two-stage measures of sex/gender had a high level of acceptance overall and that there were only a few interview drop-outs. On the basis of previous experience, the modified query can be used for further surveys, but should also be adapted in perspective. For this purpose, participatory studies are desirable that focus on how the acceptance of measures of sex/gender can be further improved and how hurtful experiences in the context of the questions asked can be avoided.

## 1. Introduction

Population-representative surveys on health are important data sources for targeting health promotion, prevention and care to specific population groups. In this way, they contribute to reducing health inequalities. You can make these, as well as the underlying mechanisms of formation, visible. The prerequisite for this is a sufficiently differentiated data base.

With regard to the standard sociodemographic variable sex/gender, a binary variable (woman/man or female/male) has been collected in population-representative surveys in Germany up to now and the results have usually been differentiated according to women and men. Both when using different questions or question modules for women and men (e.g. on gynaecological complaints) in epidemiological studies and health-related surveys and in the analysis of study results, respondent assignment was guided by a binary and cisnormative understanding of sex/gender. Cisgender means that a person identifies as a woman or a man and that this gender identity corresponds to the sex assigned at birth (Info box). The cisnormative understanding becomes apparent when, for example, the interviewer assigns a respondent to a sex/gender based on their voice in a telephone interview, or when the previous query about sex/gender did not differentiate whether it asks about official sex/gender marker, gender identity or sex characteristics [1]. Although the majority of the population is cisgender, it is scientifically, legally and ethically problematic when being cisgender is assumed to be universally valid for the entire population, as is implicit in the common binary sex/gender query.

**Info box Selected sex/gender groups [11]**

**Cisgender persons or cis** identify with the sex/gender they were assigned at birth. They describe themselves as women or as men. The definition used here does not include people who were classified as intersex at birth or who were diagnosed as intersex during the course of their lives.

Intersex, intersexual or inter* persons are born with variations of sex characteristics. They do not correspond genetically and/or anatomically and/or hormonally to the medically established norms of ‘female’ or ‘male’. This term covers a wide range of physical variations. While in some cases they are already visible at birth, others only become apparent over the course of life, e.g. during puberty, or remain unrecognised throughout life. Intersex people can have different gender identities.

Transgender, transsexual, transident or trans* persons do not or not completely identify with the sex/gender they were assigned at birth. These terms cover a variety of gender identities and expressions within and beyond the binary gender norm [32].

In everyday understanding, sex and gender are often unquestioningly equated (In German, the term ‘Geschlecht’ (sex/gender) does not differentiate between sex and gender). In contrast, a scientific distinction is made between a social (gender) and a biological (sex) dimension. Both are in complex interrelationships with each other [2–4]. The social dimension (gender) includes social norms and conventions of femininity and masculinity. In interaction with other social categories of difference (e.g. intersections with age, education), certain cultural conventions, norms, social roles and identities apply [5, 6]. Gender classifications and their intersections with other social categories of difference are linked to social power relations and the distribution of resources. On the individual level, persons can feel that they belong to one gender or to no gender (agender) in modification of and in differentiation from social norms and conventions. The biological dimension (sex) refers to genetic, anatomical and physiological, including hormonal characteristics. Both dimensions show great variations within themselves, but also in relation to each other [7–9]. Sex groups are medically defined on the basis of biological characteristics. Gender identity cannot be inferred from a person’s sex characteristics. The gender identity and sex characteristics of a person can change in the course of life (e.g. through gender reassignment procedures). This also has implications for research into health differences, which should be based on a scientifically sound definition of sex/gender.

Overlooking or denying sex/gender diversity, as implied by the sex/gender query that has been common up to now, is problematic. In the socially dominant understanding of sex/gender, innate variations in sex characteristics (intersex) are not recognised and that gender identity does not have to correspond to the sex assigned at birth (transsexuality, gender diversity) [10, 11]. The proportion of transgender and intersex persons in the population cannot yet be reliably estimated. In an international meta-analysis calculated there are 4.6 transgender people per 100,000 people [12]. In Germany, a change of official sex/gender marker and first name is possible on the basis of the Transsexual Act (TSG) from 1980. The number of these annual applications increased from 903 in 2008 to 2,687 in 2020 [13]. This increase is due, among other things, to a ruling by the Federal Constitutional Court in 2011. Until then, transgender persons had to undergo surgical sterilisation to change their personal status. With regard to intersex, a review of scientific and clinical studies estimates that between 0.018% and 2.1% or 3.8% of all births have so-called ‘variants of sex development’ or of the urogenital system [14]. The Free & Equal Initiative of the United Nations assumes that between 0.05% and 1.7% of the population are intersex [15, 16]. For a long time, intersex children were operated on after birth in order to match them to a female or male sex, so that their share of the population has probably been underestimated so far (medically not necessary surgery on children who have variations of sex characteristics and are incapable of giving consent have only been prohibited since 2021). The rough estimates indicate that minoritised sex/gender groups make up only a small proportion of the population. However, this does not justify their systematic exclusion from health studies. In terms of the public health mandate, the health situation of the entire population in its diversity should be surveyed, analysed and recommendations for action be derived from this [17, 18].

**GEDA 2019/2020-EHIS**

Fifth follow-up survey of the German Health Update

**Data holder:** Robert Koch Institute

**Objectives:** Provision of reliable information on the health status, health behaviour and health care of the population living in Germany, with the possibility of European comparisons

**Study design:** Cross-sectional telephone survey

**Population:** German-speaking population aged 15 and older living in private households that can be reached via landline or mobile phone

**Sampling:** Random sample of landline and mobile telephone numbers (dual-frame method) from the ADM sampling system (Arbeitskreis Deutscher Markt- und Sozialforschungsinstitute e.V.)

**Sample size:** 23,001 respondents

**Study period:** April 2019 to September 2020

**GEDA survey waves:**

► GEDA 2009
► GEDA 2010
► GEDA 2012
► GEDA 2014/2015-EHIS
► GEDA 2019/2020-EHIS

Further information in German is available at

www.geda-studie.de

The health situation of transgender, intersex and gender-diverse population groups is characterised by specific social challenges (e.g. discrimination, binary sex/gender and cisgender norm) and shows a particular need for action [11]. Up to now, there is only little information about the health situation of these population groups and this information mostly comes from the Anglo-American context, so that a transferability of the key figures to the German context is only possible to a limited extent. Apart from an existing need for research, the issue of sex/gender diversity is gaining political relevance due to the 2018 change of the Civil Status Act. Since then, it has been possible for intersex persons to indicate ‘diverse’ in addition to ‘female’ or ‘male’ in their sex/gender marker or to leave the field blank (the latter since 2013). Also to comply with the changed legal situation, a change in the sex/gender query is therefore required in social and health science studies.

With a modified sex/gender query, an international comparability is to be established, data gaps are to be closed and equal health opportunities for minoritised sex/gender groups are to be promoted. At first glance, the simplest solution seems to be to add ‘diverse’ to as a response category. This should give intersex persons visibility. However, the category ‘diverse’ is an officially introduced collective category that does not allow any differentiation and thus no representation of sex/gender diversity (e.g. transgender, agender and non-binary persons). This is because not all people who come into question identify themselves with this category, or they may also have a female or male sex/gender marker and therefore cannot identify with this official category. Furthermore, transgender and gender-diverse persons also change their civil status to ‘diverse’. In order to measure sex/gender diversity as accurately as possible in a scientific sense, it is not enough to introduce of the response category ‘diverse’.

In 2019, the Robert Koch Institute (RKI) launched a population-representative health survey with a modified, two-stage sex/gender query. This article describes the objectives, the procedure and the experiences with the operationalisation of sex/gender in the RKI’s study German Health Update (GEDA 2019/2020-EHIS). First, the survey of sex/gender diversity in Germany and internationally is outlined. Next, we describe the survey instrument used to measure sex/gender in GEDA 2019/2020-EHIS and the sample by sex/gender and by sociodemographic characteristics. How the sex/gender query was assessed by the interviewers and their experiences in the survey are presented in the following section. Finally, the results are summarised and conclusions and challenges are formulated.

## 2. The measure of sex/gender diversity in Germany and internationally

The spectrum of sex/gender diversity in Germany has so far been little or not at all represented in official statistics and in population-representative surveys. Many surveys either do not differentiate whether the sex/gender query refers to sex characteristics, civil status or identity. In addition, sex/gender is often recorded by an assessment of the interviewer instead of asking for it directly. This is also the case in household surveys where one person provides information about the other household members [19].

### 2.1 International examples of good practice

Internationally, there are already examples of sex/gender diversity surveys and some countries have been pioneers, including Australia. The Australian Human Rights Commission recommended in 2009 that in cases where it is necessary to collect data on the sex/gender of individuals, a further option in addition to ‘female’ or ‘male’ should be included [20]. In 2015, government guidelines followed that are intended to make transgender and gender-diverse people visible and to recognise them. In addition, they regulate the consideration of sex/gender diversity in the collection of sex/gender by government institutions (e.g. statistical data collection by ministries or in government agencies) [21]. The Australian Bureau of Statistics now collects both sex and gender identity with two separate questions and offers three response options for both (‘Male’, ‘Female’, ‘Other, please specify …’) [22].

Canada is also one of the first countries to survey both ‘sex’ and ‘gender’ beyond the sex/gender binary and cisgender norm [23]. This has also been reflected in the statistical standards since 2018, in which no specific survey instrument for sex/gender is formulated, but rather the understanding of sex/gender is presented: According to this, the biological dimension refers to the sex assigned at birth with the categories ‘male’, ‘female’ and ‘intersex’ [24]. ‘Gender’ refers to the gender identity and/or the gender that a person expresses in their daily life (gender expression), regardless of gender identity, with the categories: ‘Man’, ‘Woman’, ‘Non-binary person’ [25]. It is also recognised that some persons do not identify with a particular gender and that of the gender identity and/or gender expression can change throughout the life course. Some surveys are planned to ask only about ‘gender’, while others will continue to use the two-step approach [26].

An example of recommendations from academia for surveying sex/gender diversity in population-based studies is the 2014 Gender Identity in U.S. Surveillance Group (GenIUSS Group) [27]. For surveys of the general population, they recommend a two-step query to include transgender persons and other minoritised sex/gender groups: The self-reported records of sex assigned at birth (‘Male’, ‘Female’) and current gender identity (‘Male’, ‘Female’, ‘Transgender’, ‘Do not identify as female, male, or transgender’).

An analysis of different two-stage sex/gender survey instruments used in population-representative studies in the USA and Canada problematises the response option ‘transgender’ when asking about gender identity, since persons do not necessarily identify as transgender [28]. A two-to three-step questionnaire is recommended, which should first ask about the sex entered on the birth certificate (‘Male’, ‘Female’), and then about the current gender identity (‘Male’, ‘Female’, ‘Indigenous or other cultural gender minority identity (e.g. two-spirit)’, ‘Something else (e.g. gender fluid, non-binary’). A third (filter) question should be asked if respondents have chosen a different option for their gender identity than is recorded on their birth certificate. This asks about the gender that is lived in everyday life (‘Male’, ‘Female’, ‘Sometimes male, sometimes female’, ‘Something other than male or female’). However, transferability and general comprehensibility in the German context is limited. Nevertheless, the analysis provides important information for the development and reflection of the survey instrument presented here.

### 2.2 Developments in Germany

In Germany, efforts are being made both by public institutions and by academics to measure sex/gender diversity in a differentiated way. For example, in 2018, the Federal Anti-Discrimination Agency – which was established after the introduction of the General Equal Treatment Act (AGG) in 2006 – commissioned an expertise on the topic of discrimination in social science repeated surveys in Germany (e.g. in the Microcensus and the Socio-Economic Panel) [19]. Here the focus on the categories protected by the AGG was sex/gender, ethnic origin/ racializing ascriptions, religion/belief, disability/impairment, age and sexual orientation. In addition, the report also makes recommendations for repeated surveys. According to these, the question on gender identity should be covered by a question with at least four possible answers: ‘Which of the following terms to describe sex/gender applies to you? (Response options: ‘Female’, ‘Male’, ‘Transman’, ‘Transwoman’, ‘Trans* (e.g. transgender, transident, transgender, transsexual)’, ‘Inter* (e.g. intersex, inter sex/gender, between genders), ‘Different, namely…’, ‘For me personally, I reject classification into sex/gender categories’. The third to sixth answer categories are considered optional or they can be given as a further differentiation of the category ‘Different, and that is…’ Subsequently, the sex assigned at birth should be asked.

Since the 1970s, recommendations for collecting central sociodemographic characteristics in surveys have been available at irregular intervals (so-called demographic standards) [28]. The aim is to standardise the sociostructural survey characteristics in population surveys in order to enable greater comparability between individual surveys. However, there are currently no agreed minimum requirements for standard items and standard variables, such as those on sex/gender.

Since 2020, the Consortium for the Social, Behavioural, Educational and Economic Sciences (KonsortSWD) has had the task of further developing and harmonising the research data infrastructure in Germany. In this context, the measurement of sex/gender in survey studies is also addressed. For this purpose, an overview of the survey instruments of sociodemographic variables in large German studies was developed and the challenges of harmonisation were described [29]. For the survey of sex/gender, the conclusion is drawn that since the introduction of the sex/gender entry ‘diverse’, sex/gender diversity has been increasingly taken into account in the various survey instruments, but due to the diversity of survey instruments, less comparability is possible. The survey of gender identity is seen as a useful addition, even if this characteristic is not (yet) one of the standard demographic variables.

As one of the first nationally representative studies, the study Health and Sexuality in Germany (GeSID) took up the recommendations of the GenIUSS Group [27] and asked all participants about their sex assigned at birth (‘Male’, ‘Female’) and about their gender identity at the time of the survey (‘Male’, ‘Female’, ‘Trans*/Transsexual’, ‘Neither female, male nor trans*/transsexual, but’) [30].

In the questionnaire of the Socio-Economic Panel (SOEP) of Sample Q (LGB), a two-stage sex/gender query was used in 2019, which first asks about the sex entered on the birth certificate at birth (‘Male’, ‘Female’) and then about gender identity (‘Male’, ‘Female’, ‘Transgender’) [31]. In the meantime, an adapted two-stage questionnaire has been in use in the SOEP questionnaire since 2022. In addition to ‘male’ and ‘female’, the question on gender identity now contains an open response option ‘Other gender not listed here and namely:’.

In an interdisciplinary joint project funded by the Federal Ministry of Health, a toolbox for operationalising sex/gender diversity in research on health care, health promotion and prevention is currently being developed (duration 05/2020 to 06/2023) (DIVERGesTOOL). The aim is to develop a generally usable set of questions for the sex/gender query in epidemiological health studies and to additionally offer instruments for the consideration of specific study populations and questions. This should enable or facilitate the integration of the different dimensions of sex/gender as well as their complexity, interdependence and mutual influence in health research.

## 3. The operationalisation of sex/gender

The measure of sex/gender in the study German Health Update (GEDA 2019/2020-EHIS) is intended to meet several requirements: First, a theoretically sound definition of sex/gender is used and operationalised in the survey instrument. Secondly, the sex/gender survey builds on experiences with already internationally established survey instruments. Thirdly, a binary evaluation option is retained in order to maintain continuity with previous surveys and to enable weighting according to the data from the Federal Statistical Office [3, 11].

Based on international experience, a survey instrument was developed that operationalises the sex and gender in a two-stage query as follows:

**Which sex (German: Geschlecht) was entered on your birth certificate at birth?**

1. Male
2. Female

**Which gender (German: Geschlecht) do you feel you belong to?**

1. Male
2. Female
3. Or another, namely: …

The biological dimension is measured by the sex entered on the birth certificate at birth. This is based on a medical classification according to externally visible sexual organs and does not take into account any further sex characteristics. The sex marker can therefore differ from the biological sex. Intersex traits of a person might not be diagnosed or is only diagnosed in the course of life or this was diagnosed at birth, but no other sex/gender marker than ‘female’ or ‘male’ was available or another entry was not selected. Since the GEDA survey includes persons from the age of 15, the last aspect was not relevant (only since 2013 has it been possible to leave the sex/gender marker open, or only since 2018 has it been possible to enter ‘diverse’ as the sex/gender marker). In this sense, the operationalisation of the biological dimension (sex) perpetuates an official misattribution of intersex people. Nevertheless, this query was chosen in order to maintain a binary response category and thus allow the variable to be weighted according to the data from the Federal Statistical Office.

Since a person does not have to identify with the sex/gender assigned at birth, or not completely, gender identity was measured as an aspect of the social dimension in a second step. A person can identify with no gender or a gender other than the one assigned to them at birth. In addition to ‘female’ and ‘male’, a third, open response option was provided. Although the formulation ‘or (please elaborate):’ instead of ‘or another, namely:’ was discussed in order to avoid othering of further gender identities. However, this variant was discarded in order to achieve better comprehensibility in the oral questionnaire and consistency with other survey instruments.

By combining both questions, cisgender and transgender as well as gender-diverse people can be identified. For example, if ‘male’ is given for sex at birth and ‘female’ for gender identity, the respondent is classified as a transgender woman. This does not necessarily correspond to the identity of a specific person, but is a categorization in order to be able to make a statistical analysis. Another misattribution may be included in this categorisation, as adult intersex persons have ‘female’ or ‘male’ entered on their birth certificate respectively, and are therefore described as transgender when they identify with an opposite gender. Intersex people can also be in the cisgender group if they identify with the sex/gender they were assigned at birth, or in the gender-diverse group if they do not identify as female or male. Especially with regard to intersex people, the survey instrument proves to be too undifferentiated. If a identity such as ‘non-binary’ was chosen in the third answer option in the question about gender identity, this entry was assigned to the category ‘gender-diverse’. The category ‘gender-diverse’ is therefore no longer a self-description of the respective person, but a grouping of very different gender identities. This grouping was chosen in order to be able to reach a statistically relevant size.

## 4. Sample description

The statistical analyses in this article serve solely to describe the sample composition according to the sex assigned at birth and the gender identity after the introduction of the new two-stage measures of sex/gender. This description is further differentiated according to age and other socioeconomic and social characteristics (educational and employment status, equivalent income, partnership, marital status) [33, 34]. Further statements on different sex/gender groups are not made. Therefore, all analyses were carried out without sample weighting. The methodology of the GEDA 2019/2020-EHIS survey has already been described in detail elsewhere [33].

A total of 23,001 respondents participated in the GEDA 2019/2020-EHIS survey. The response rate was 21.6% according to the standards of the American Association for Public Opinion Research (AAPOR) [33]. Of these, 52.65% were recorded as ‘female’ at birth and 47.35% as ‘male’. 52.30% of the respondents identified with their sex assigned at birth as ‘female’. These individuals can be described as cisgender women. 46.94% of the respondents are considered cisgender men. There were 0.62% of respondents who provided information indicating that they are not cisgender. Among these, 0.13% have not identified themselves as either male or female group and are referred to in the study as ‘gender-diverse’ persons. 0.49% identify as women or men respectively, although they were assigned a different sex at birth. These respondents are referred to in the study as transgender persons. Of these, 0.18% are transgender men (male identity and assigned female at birth), 0.31% are transgender women (female identity and assigned male at birth). In contrast to the measure of sex assigned at birth, there are a few missing data (‘don’t know’ or ‘no data’) in the survey of gender identity. In relation to the total sample, these are 33 respondents (0.14%) (Table 1). The instrument for the two-stage survey of sex/gender was also used in the ‘Study on head, back and neck pain in Germany (2019/2020)’ conducted at the RKI almost at the same time, with comparable methodology but a significantly smaller number of cases [35]. The determined proportions of cisgender, gender-diverse and transgender persons as well as the proportion of missing values are almost identical (Annex Table 1).

The sample composition of cisgender and transgender as well as gender-diverse persons partly shows pronounced differences. In terms of age distribution, the subsamples of cisgender and transgender women differ only slightly from each other. Transgender men have a higher proportion of younger persons than cisgender men. Particularly gender-diverse respondents are significantly younger than the general population. About 51.7% of the persons in question are between 18 and 39 years old, compared to only 20.9% in the overall sample. In the survey, there is a tendency for transgender and gender-diverse people to have a low level of education and income more often than cisgender people. The connection with education is particularly pronounced among transgender men, and that with income among gender-diverse people. Particularly transgender women are less likely to be employed than cisgender women, but gender-diverse persons are also proportionally less likely to be employed compared to the total sample. With regard to a stable partnership, there are no marked differences between cisgender and transgender or gender-diverse persons between cisgender and transgender women. In contrast, transgender men live in a partnership less often than cisgender men. The proportion of respondents in a stable partnership is lowest among gender-diverse persons. Gender-diverse persons in particular are more likely to have a single marital status. Gender-diverse people are 61.1% single and 19.4% married in contrast to 24.5% single and 54.6% married in the total sample. However, transgender women and men are also less often married and slightly more often divorced than cisgender women and men (Table 2). The differences described cannot be generalised due to the sample size and are probably also partly due to the younger age of the transgender and gender diverse sub-samples.

## 5. Survey of the interviewers

In order to ascertain the acceptability of the two-part sex/gender query, a process data analysis was conducted in November 2020 to record interview dropout rates, as well as a written survey of people who had conducted interviews in GEDA 2019/2020-EHIS. The questionnaire was sent to the 90 interviewers in November 2020. 42 interviewers (46.7%) participated in the survey by the end of November. The 42 interviewers conducted approximately 7,000 of the total 23,124 GEDA interviews. The written information provided by the interviewers was analysed quantitatively with descriptive statistics and via a summary content analysis according to Mayring [36] with a quantification of the categories. Selected citations are presented as examples (Table 3).

### 5.1 Dropout rates and reported reactions of the interviewees

For the process data analysis, the GEDA data set was prepared and then analysed with the statistics programme STATA version 17.0. The analyses included descriptive frequency counts of the dropouts by the interviewees at the last telephone interview contact.

**Figure 1:**
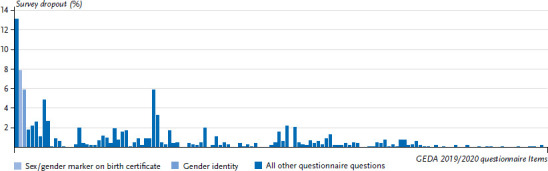
Terminations of the survey by the interviewees differentiated according to the questions of the entire GEDA 2019/2020-EHIS questionnaire (n=1,056 terminations by the respondents) Source: GEDA 2019/2020-EHIS

In total, there were 1,056 interview terminations by the interviewees. In relation to the total number of complete interviews conducted for GEDA 2019/2020-EHIS, the number of dropouts by interviewees is very low. 13.8% of interview dropouts by respondents occurred at the two-step sex/gender query. This corresponds to 83 terminations after the question about the sex registered at birth and 62 terminations after the question about gender identity. These terminations are final terminations, after which further attempts to call the interviewee did not result to an interview with the person questioned. If one looks at the dropout rates in an overview of the entire questionnaire, it becomes apparent that the beginning of the questionnaire is characterised by many interview dropouts. Thus, the sex/gender query, which follows directly after the consent to participate as the second and third question in the questionnaire, is also characterised by many dropouts (Figure 1). Accordingly, the relatively high dropout figures for the sex/gender question cannot be attributed exclusively to its content, but also to its positions at the beginning of the questionnaire.

The interviewers described different reactions of the interviewees when asked about the sex registered at birth and the gender identity. It is not possible to reconstruct how often irritation or acceptance of the query occurred per interviewer. While 26 interviewers reported neutral and accepting reactions to the question about the sex registered at birth and eight interviewers reported negative reactions (Table 3, Citation 1, 2) 14 interviewers reported neutral and accepting reactions to the question about gender identity and 29 reported negative reactions (Citation 3). Four interviewees reported that younger respondents and five interviewees that women showed more acceptance and less irritated reactions to the sex/gender query (Citation 4). For older respondents, 19 interviewers described angry and irritated reactions, scepticism and interview drop-outs (Citation 5). This was reported by five interviewers, especially for older men as opposed to older women (Citation 6).

Nine interviewers stated that the respondents had problems understanding when asked about the sex assigned at birth and 25 interviewees when asked about their gender identity. Interviewers reported comprehension problems especially for the term ‘birth certificate’ among respondents whom they perceived as ‘of non-German origin’ and among younger persons (Citation 7, 8). When asked about gender identity, nine interviewees described that there was confusion with sexual orientation probably due to the wording of the question about ‘belonging to a gender’ (Citation 9, 10). Five interviewees reported that they included further explanations and repetitions of the question of gender identity (Citation 11).

### 5.2 Attitude of the interviewers

18 interviewees described a neutral attitude towards the implementation of the two-step sex/gender query. The interviewers tended to regard the question about gender identity as significantly less meaningful than the question about the sex registered at birth. This assessment correlated with a higher age of the interviewers (p=0.007), a connection that was not seen for the question about the sex marker at birth (p=0.850). Seven out of 42 interviewers were uncomfortable asking these questions because the sex/gender query might seem redundant to the respondents. The background to this is that the sex/gender of the respondents had already been asked beforehand by means of the Kish-Selection-Grid (procedure for random selection of respondents in households with several persons) or because the sex/gender should already be recognisable from the voice in the view of the interviewers (Table 3, Citations 11, 12, 13). Single interviewers reported that they deviated from the given standardisation of the questionnaire in order not to have to give further explanations of the two-step sex/gender query (Citation 14).

## 6. Discussion and outlook

The aim of this article was to describe the introduction of a new two-step sex/gender questionnaire in the RKI’s GEDA 2019/2020-EHIS study, which distinguishes between sex assigned at birth and gender identity. In addition, respondents were provided with an open response option regarding gender identity. Overall, the two-stage measures of sex/gender has proven to be functional and easy to implement. Out of 23,001 respondents, 22,826 persons are classified as cisgender, 113 persons as transgender and 29 persons as gender-diverse. 33 respondents had missing information regarding gender identity. In another study by the RKI with a comparable procedure, the proportions were very similar. This indicates a high reliability of the measurement instrument.

In relation to the total number of interviews conducted, the number of terminated interviews for the two-stage measures of sex/gender is very low, and the survey of the interviewers shows a high acceptance of this questionnaire among the interviewers and the respondents overall. The interviewers reported that younger respondents and women showed more acceptance and less irritated reactions than other groups when asked about gender identity. Problems of understanding the term ‘birth certificate’ were reported among younger respondents and people with a presumed migration background. Furthermore, the question about gender identity was occasionally confused with sexual orientation. There was a need for training for the interviewers on the background and objectives on the measures of sex/gender and its standardisation. In addition, further explanations should be integrated into the questionnaire.

An obstacle for the measure of sex/gender – especially for non-binary persons – is the Kish-Selection-Grid used for the selection of respondents. This contains a binary measure of sex/gender and is used by the interviewers before the actual interview to identify the person to be interviewed within the household. Against this background, it should be examined in future whether a variant of the Kish-Selection-Grid can be used in which sex/gender is not asked.

When examining the composition of the sample according to socio-demographic characteristics, some differences are noticeable. In particular, gender-diverse persons are younger than cisgender and transgender respondents. In addition, transgender and gender-diverse persons are more often not employed, more often have a lower education and a lower income, live less often in stable partnerships and are more often single and less often married. However, the findings on the composition of the sample should be interpreted with caution. For example, low education can largely be explained by a younger age in the concerned groups and having not yet completed vocational training. A more robust analysis of such correlations should be carried out on the basis of larger samples, for example with the help of pooled survey waves. Furthermore, own studies on the health of minoritised sex/gender groups are useful, which should be co-designed, conducted and accompanied by community members.

The survey instrument enables respondents to situate themselves beyond the binary sex/gender and cisgender norm and thus acknowledges the sex/gender diversity. It should be noted that in the survey used here, intersex people cannot assign themselves according to their sex characteristics with the indicator of the sex registered at birth. It is true that since 2013 the sex/gender marker can be left open and since 2018 it can be indicated as ‘diverse’. However, since this was not possible at all for a long time, a separate question would have to be inserted in surveys to record intersex or another indicator would have to be used to operationalise sex.

Furthermore, very different sex/gender groups are summarised in the category ‘gender-diverse’. This thus becomes a collective category, which, also due to the small number of cases, can no longer make differences within this category visible. In addition, the coding of the open response category and the assignment of respondents as transgender based on different information in the two-stage measures of sex/gender can be problematic, since third-party attributions take place here. This problem should be discussed with community members in particular.

In the current and future GEDA analyses that are carried out and published, gender identity is used as a binary variable (female/male), so that transgender and cisgender people are analysed together. Gender-diverse people are not shown separately due to the small number of cases, but remain included in the category of all respondents as a whole. This procedure is intended to recognise the gender identity of transgender persons. Besides a possible misattribution of sex/gender, however, it is problematic that this approach can no longer show that transgender have very different health opportunities compared to cisgender people [11].

An open question is how different questions about sex-related physical differences can be used when study participants are not cisgender. In this context, sex/gender serves as a filter variable. Respondents should be free to choose which questionnaire they want to fill out. In order to avoid hurtful experiences through use of insensitive language for these study participants, it would be appropriate to offer survey instruments for further sex/gender groups in addition to those for women and men.

The available data show that people also participate in survey studies for whom the sex assigned at birth and the gender identity do not match. These should be given the opportunity to express their sex/gender in surveys, which in perspective will also improve the possibilities for researching the connection between sex/gender diversity and health. In order to achieve better acceptance and minimise hurtful experiences (e.g. dysphoria), participatory studies to further develop the survey instrument are desirable. For example, the question about the sex assigned at birth can be experienced as hurtful [37]. When making adjustments, however, the general comprehensibility and acceptance of the survey instrument must also be ensured. Valuable information for the further development and harmonisation of measures of sex/gender can also come from the DIVERGesTOOL project, from studies on the health of transgender and intersex people as well as non-binary people (TASG, InTraHealth). The experiences reported here with the modified measures of sex/gender are therefore intended to contribute to the debate about the increased consideration of sex/gender diversity in health studies. This includes the careful further development of the instruments used on the basis of these and future experiences.

## Key statement

In the sense of public health, the health situation of the entire population should be recorded in its diversity, evaluated and recommendations for action derived from this.

The survey of sex/gender diversity should not be limited to the introduction of the response category’ diverse’.

The survey instrument used enables respondents to situate themselves beyond the binary sex/gender and cisgender norm and thus acknowledges sex/gender diversity.

Overall, the two-stage query of sex/gender has proven to be functional and easy to implement.

Further discussions with representatives of transgender, intersex and gender-diverse people are helpful to further develop the query to meet the needs of large health studies as well as minoritised sex/gender groups.

## Figures and Tables

**Table 1:**
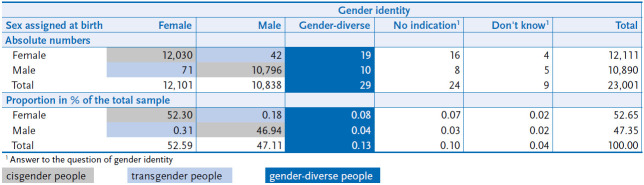
Sex entry at birth and gender identity in absolute numbers and sex/gender in the total sample (n=23,001) Source: GEDA 2019/2020-EHIS

**Table 2:** Sex/gender groups according to socidemographic factors, proportion in % (cisgender women n=12,030, cisgender men n=10,796, transgender women n=71, transgender Men n=42, gender-diverse persons n=29), Source: GEDA 2019/2020-EHIS [33]

Female (cis)		Female (trans)	Male (cis)	Male (trans)	Gender-diverse	Total
**Age group**						
18–39 years	18,31	19,12	23,78	30,95	51,72	20,93
40–59 years	28,17	27,94	33,00	30,95	24,14	34,02
≥60 years	53,52	52,94	43,22	38,10	24,14	45,05
**Education**						
Low education group	8,19	12,68	5,65	23,81	17,24	7,05
Medium education group	48,54	46,48	35,14	45,24	27,59	42,20
High education group	43,27	40,85	59,21	30,95	55,17	50,75
**Employment status**						
Employed	50,40	37,14	56,62	50,00	44,83	53,27
Not gainfully employed	49,60	62,86	43,38	50,00	55,17	46,73
**Equivalent income**						
1. Quintile	13,41	26,76	10,79	26,83	41,38	12,28
2.–4. Quintile	61,44	60,56	54,93	43,90	41,38	58,33
5. Quintile	25,15	12,68	34,28	29,27	17,24	29,39
**Stable partnership**						
Yes	64,68	60,56	73,18	59,52	46,43	68,63
No	35,32	39,44	26,82	40,48	53,57	31,37
**Marital status**						
Unmarried	20,64	32,39	28,59	28,57	64,29	24,48
Married	52,04	46,48	57,60	42,86	17,86	54,57
Widowed	15,96	5,63	6,06	16,67	14,29	11,27
Divorced	11,37	15,49	7,76	11,90	3,57	9,68

**Table 3:** Selected citations from the written questionnaire of the interviewers Source: GEDA 2019/2020-EHIS

**On the reactions of the interviewees**
1: “Usually problem-free response.” (I 20)
2: “One said straight away not so and hung up – Otherwise there were no particular reactions.” (I 26)
3: “The participants reacted mostly angrily, without understanding and sometimes aggressively. Many interviews were ended at this point by the participants hanging up.” (I 3)
4: “Women had more humour and understanding than men.” (I 35)
5: “More scepticism among the older ones. ‘This is such a modern issue.’“ (I 20)
6: “Rather older men who tended to feel irritated by the question about their gender identity (possibly questioned in their masculinity).” (I 16)
**On problems of understanding**
7: “Persons of non-German origin often didn’t know what to do with ‘birth certificate’.” (I 13)
8: “Especially younger persons who probably never needed their birth certificate before. Answer: ‘don’t know.’“ (I 34)
9: “Some even started talking about their sexuality such as: ‘How? I’m not gay!’“ (I 39)
10: “Some have confused belonging with being attracted to a sex/gender.” (I 12)
**On the attitude of the interviewers themselves**
11: “What is the point of the question *(Note: Meant is the question about gender) –* you can tell.” (I 24)
12: “Question ‘considered superfluous’ because sex/gender is recognisable from voice.” (I 10)
13: “Perhaps irritated because sex/gender was already asked via the Kish-Selection-Grid.” (I 41)
14: “Added the sentence ‘then there is a supplementary question’ after the question about sex.” (I 31)

I=Interviewer

Editor’s comment: The spelling of written citations has been adjusted and abbreviations written out.

**Annex Table 1:**
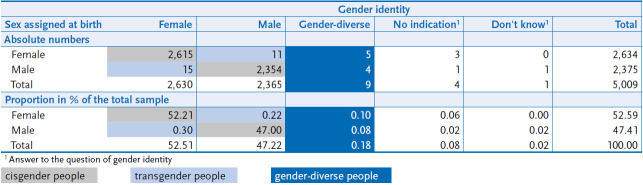
Sex entry at birth and gender identity in absolute numbers and sex/gender in the total sample (n=5,009) Source: Study on head, back and neck pain in Germany (2019/2020)
